# Immune Response to a Variable Pathogen: A Stochastic Model with Two Interlocked Darwinian Entities

**DOI:** 10.1155/2012/784512

**Published:** 2012-12-02

**Authors:** Christoph Kuhn

**Affiliations:** Biomedical Optics Research Laboratory, Clinic of Neonatology, University Hospital Zürich, Frauenklinikstrasse 10, CH-8091 Zürich, Switzerland

## Abstract

This paper presents the modeling of a host immune system, more precisely the immune effector cell and immune memory cell population, and its interaction with an invading pathogen population. It will tackle two issues of interest; on the one hand, in defining a stochastic model accounting for the inherent nature of organisms in population dynamics, namely multiplication with mutation and selection; on the other hand, in providing a description of pathogens that may vary their antigens through mutations during infection of the host. Unlike most of the literature, which models the dynamics with first-order differential equations, this paper proposes a Galton-Watson type branching process to describe stochastically by whole distributions the population dynamics of pathogens and immune cells. In the first model case, the pathogen of a given type is either eradicated or shows oscillatory chronic response. In the second model case, the pathogen shows variational behavior changing its antigen resulting in a prolonged immune reaction.

## 1. Introducing Relevant Prior Knowledge

### 1.1. Putting the Objectives of the Paper into Context

 The wide relevance of pathogens, such as the influenza virus, the human immunodeficiency virus (HIV), or trypanosomes, give great significance to those studies, where pathogens are able to vary their antigens while still vital in the host and where the host's immune system mounts specific immune reactions (by clonal selection, somatic hyper-mutation, and forming an immune memory) [1]. 

Investigations of long-term dynamics of hosts and their immune systems in environments that consist of variable pathogen strains are especially valuable in, first, knowing how duration of the immunological memory can influence the pathogen competition and in, second, evaluating whether the pathogen can be a selective force that can shape the evolution of the immunological memory [2]. The study of these processes is, however, a very complex endeavor. Indeed, in the lowest approximation of understanding the interaction between the invading pathogen and the immune system, the selected immune clones do not go on to future generations of the infected host. Moreover, the ability of a virus/bacteria to survive within the host does not necessarily imply good ability to infect other hosts, and thus survive and evolve. 

In this paper, we will focus solely on modeling the dynamics of an infection within one host, and we will provide possible understanding of how the pathogen load and pathogen diversity influence the immune response [3–5]. Can the complex process of an immune response be simplified to be tractable theoretically but still represent some basic facts from immunobiology [6]? In understanding the immune response, it is well established that both the pathogen [7] invading the host as well as the effector [8, 9] of the host's immune system (trying to get rid of the pathogen) undergo a step-by-step Darwinian process, namely, multiplication with mutation, and selection. This process is stochastic in nature: chance events weighted by fitness influence the processes of multiplication, mutation and selection. The immune response involves two such entities, which are coupled: the pathogen, that is, virus, bacterium or parasite, on the one hand, and the immune effector cell together with its immune memory cell as idioblast on the other hand. The specific immune response to the pathogen worsens the conditions for the pathogen to thrive, and ultimately eliminates the pathogen, at best, without harming the host. 

In the following section (Section 1.2), we provide a short description of the basic biological facts of an immune response as well as some mathematical background on continuous models studied previously in theoretical immunology (Section 1.3). We then propose on grounds of a simple stochastic approach of a Darwinian entity (Sections 2.1–2.4), a stochastic model of an immune response (Section 3.1) by coupling two Darwinian entities. We apply this model to a nonvarying pathogen (Section 3.2), and to the challenging problem of a variable pathogen (Section 3.3), for example, a strain of a pathogen transforming into another strain each with different antigens that are presented to the immune system. Finally we model the maturation process from a naive immune cell to an effector cell that contributes to the elimination of the pathogen (Section 4). 

### 1.2. Basic Facts from Immunology and the Request for a Simple Model

The interaction between a pathogen, which can be a virus, a bacterium or a parasite that has invaded a host, and the reaction of the host's immune system, which is a concerted action of multiple players in time and space, is certainly not simple [1]. It includes the fully developed specific adaptive/acquired immune system, mainly the B and T lymphocytes as well as the innate immune system, mainly the macrophages, which are dumping cells, and the soluble cytokines, which themselves have a wide spectrum of biological activities that help to coordinate the complex immune regulation. 

An important part of the specific adaptive/acquired immune system is the “endogenous-cellular” path, where the pathogen—which is usually a virus, but it can also be an intracellular bacteria—proliferates within the cytosol of the host cell. The antigens of this pathogen via proteasome, endoplasmatic reticulum and Golgi apparatus are presented at the surface of this cell by the major histocompatibility complex I (MHC-I). If such a cell happens to be a dendritic cell (DC), which is an antigen-presenting cell (APC) that transports the antigen from its entrance site to the corresponding secondary lymph organ, the antigen presented can be recognized specifically by the antigen receptor (CD8) of a “matured T lymphocyte” that entered the lymphatic system. Before naive T lymphocyte have undergone maturation: first, a naïve T lymphocyte in bone marrow or thymus undergoes T-cell receptor rearrangement (*β* selection). T cells with high affinity to self-peptides MHC are eliminated (negative selection), whereas T cells with T-cell receptors that are able to bind self-peptides MHC molecules with at least a weak affinity survive (positive selection) and circulate in the peripheral lymphatic system. The matured T lymphocyte, recognizing the antigen by high affinity to the antigen-loaded MHC, transforms into an effector cell and proliferates. These cells are short-lived and some participate in forming memory cells. The cytotoxic T-lymphocytes (CTL) then only kill those cells, which harbor the pathogen by recognizing its antigens presented at the surface of the infected cell by MHC-I molecules. Thus, further proliferation of the pathogen is diminished. Viruses are intracellular parasites that depend on the host cell to survive and replicate. The host cell can be damaged either directly by the virus or by the immune response it provoked consisting of cytokines, macrophages, and antibodies and, most important, the CTLs. The balance of good or bad harm depends on the virus lethality, the amount of virus present (virus load), the amount of tissue infected (cyto-pathogenicity) and the affinity of CTL-response, and duration of CTL response (chronicity of the infection [10]).

One can note another path, the “exogenous-humoral” path whereby the pathogen, which is usually a bacterium, but it can be a virus or a parasite as well, proliferates in the extra-cellular space of the host. The pathogen, or fragments of it, is endocytosed into the phagolysosome of a host's APC, which transports the antigen as a DC to the secondary lymph organ, and the antigens of the pathogen are presented at the surface of this cell by MHC-II molecules. The antigen presented can be recognized specifically by the antigen receptor of a matured helper T lymphocyte (called CD4 Th1 and CD4 Th2, resp.). A matured B lymphocyte (interacting specifically with the matured helper T lymphocyte) becomes activated (transforms into an effector cell and proliferates: these cells are short-lived, and some participate in forming memory cells), it is then called PC- (plasma-cell) producing antigen receptors (called IgG and IgE, resp.) which are soluble. These antibodies, or immune globulins, mark the pathogen, which in turn is phagozytosed and killed by macrophages.

For the function of specific adaptive/acquired immune system, the B-cell and T-cell memory is essential [11–14]. The immune memory renders the immune response at multiple encounters with the same pathogen more efficient than at the first encounter. 

The mathematical model in Section 3 considers only the effector cell properties (i.e., proliferation, cell death and memory cell formation) of the immune system and the pathogen properties (i.e., proliferation, cell death and variation) thus justifying the applicability of same conceptual frame of a Darwinian entity. 

### 1.3. Previous Mathematical Models of the Immune Response

Previous approaches on the theoretical understanding of the interaction between an invading pathogen and the host's immune system [15–23], especially on the issue of multistrain pathogens [24–27], are derived from deterministic models and are continuous in time. Continuous models provide a good representation of the dynamics when there are many participants and when fluctuations are small. These models are based on establishing a reasonable set of first-order differential equations that are assumed to be generic equations describing the properties of single cells [20]. The rates of change with respect to time of each variable describing the mean values of fractions of a total cell population are equal to a corresponding source (replication rate) and sink (death rate and rate at which new strains are generated). One studies, respectively, analytic and numerical solutions, which have mainly nonlinear properties. In the most suitable example [3, 4], these authors introduce the following differential equations with five variables as follows:

(1) $$\begin{array}{c} \dot{x}=\lambda -\left(d-\beta v\right)x,\\ \dot{y}=\beta xv-\left(a+pz\right)y,\\ \dot{v}=ky-uv,\\ \dot{w}=\left(cy-fy-r\right)w,\\ \dot{z}=fyw-bz, \end{array}$$

where, *x* represents the uninfected host cell, which proliferates (rate *λ*), dies (rate *dx*), and gets infected (rate *βvx*), *y* represents the infected host cell, which has been infected (rate *βxv*) and dies (rates *ay* and *pzy*), *v* represents the free virus, which proliferates within infected host cell followed by expulsion (rate *ky*) and declines (rate *uv*), *w* represents the immune precursor/memory-cell, which proliferates (rate *cyw*), differentiates into immune effector cell upon antigenic challenge (rate *fyw*), and dies (rate *rw*), *z* represents the immune effector cell, which has differentiated from immune precursor/memory-cell (rate *fyw*) and dies (rate *bz*). 

The authors [3, 4] give parameter regions of their model, for example, the case of low virus load, where the immune system is nonresponsive, the case of high load of noncytopathic virus, where exhaustion of the immune system occurs, and the case of immune memory function where the immune response is persistent. They apply the model successfully to infections with the Lymphocyte Choriomeningitis virus (LCMV) and the HIV. Another Ansatz related to antigenic variation is given by [5]

(2) $$\begin{array}{c} {\dot{v}}_{ij}=\left(r_{ij}-p_{i}x_{i}-q_{j}y_{j}\right)v_{ij},\\ {\dot{x}}_{i}=\eta c_{i}\sum_{j}v_{ij}+\left(c_{i}\sum_{j}v_{ij}-b\right)x_{i},\\ {\dot{y}}_{j}=\eta k_{j}\sum_{j}v_{ij}+\left(k_{j}\sum_{j}v_{ij}-b\right)y_{i}, \end{array}$$

where, *v*_*ij*_ represents the virus variants with sequence *i* in epitope *A* and sequence *j* in epitope *B*, both coexistent, which proliferate (rate *r*_*ij*_) and being killed by CTLs (rates *p*_*i*_ *x*_*i*_ *v*_*ij*_ and *q*_*j*_ *y*_*j*_ *v*_*ij*_), *x*_*i*_ represents the CTLs against sequence *i* of epitope *A*, which proliferate upon activation or being already active (rates *ηc*_*i*_∑_*j*_ *v*_*ij*_ or *c*_*i*_(∑_*j*_ *v*_*ij*_)*x*_*i*_) and die (rate *bx*_*i*_), *y*_*j*_ represents the CTL against sequence *j* of epitope *B*, which proliferate upon activation or being already active (rates *ηk*_*j*_∑_*j*_ *v*_*ij*_ or *k*_*j*_(∑_*j*_ *v*_*ij*_)*y*_*j*_) and die (rate *by*_*j*_).

These coupled nonlinear differential equations investigate the complex phenomena occurring in a host which is infected by a heterogeneous pathogen population, namely, inducing a fluctuating immune response against multiple epitopes with the potential of a shift of immunodominance by escape in one epitope (for a simple case the options are termed *A*_1_, *B*_1_, *C*_2_, and *D*_2_ with sequences *A* and *B* at epitope 1 and sequences *C* and *D* at epitope 2, resp.). 

Systems (which would die according to their differential equations approximation), when taking into account the discrete character of their microscopic components, display the emergence of macroscopic localized subpopulations with collective adaptive properties that allow their survival and development [28–30]. Simulations based on a hybrid model generate a more faithful approximation of the reality of the immune system [31].

## 2. Developing the Methods

### 2.1. Modeling a Darwinian Entity

**Figure 1:**
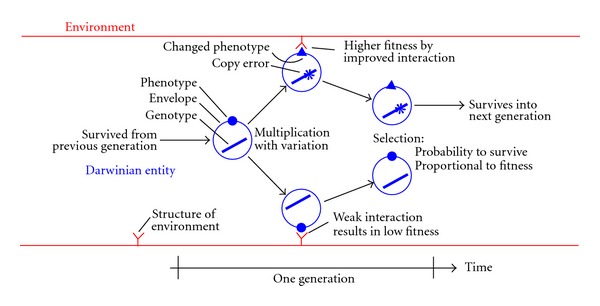
Schema of a Darwinian entity. An individual is singled out from the population. A period of one generation is shown. Incidental copying error occurs during multiplication (changed genotype resulting in changed phenotype) with new fitness in reference to its structured environment. The probability of being selected to survive is given according to new fitness.

Within the schema of general evolutionary biology, an entity, and thus its clonal population of individuals, undergoes a step-by-step Darwinian process from one generation to the next, that is, multiplication with random mutations and selection biased by fitness in the dependency to the actual environment (Figure 1). Each entity carries an information storage device (genotype), for example, a polymer (i.e., DNA or RNA) with a specific monomer sequence, which, in the multiplication phase, is copied with occasional mismatches (copying error probability per monomer). In the selection phase, each individual entity has a certain probability to be selected to survive according to the fitness of the phenotype (retrieved from the information storage device) in reference to its environment. Many and sustained step-by-step Darwinian processes are required from the first replicating molecule up to the emergence of mankind and many species emerged and others became extinct along the long way called Darwinian evolution.

Biological conduct is immanently stochastic, especially in the view of a cell population dynamics following a step-by-step Darwinian process. Stochastic models offer the benefit of handling the dynamics of whole population distributions (with their mean and standard deviation as deduction). These models provide a good representation of the dynamics when the numbers of participants in the process are small or when fluctuations are large. (e.g., extinction or initiation of infection). It is also worth noting that for studying extinction probabilities, it is natural to turn to stochastic models. 

Some stochastic approaches deal with birth-death processes by solving “Master equations” [32], by discrete-time multitype branching processes [33, 34], and by modeling gene-amplification process with branching random walks [35, 36]. Our approach in this paper is based on the theory of branching processes, more precisely on some multitype modifications of the standard Galton-Watson processes examined in detail [37–41]. 

### 2.2. Dynamical Stochastic Process of an Entity with Multiplication and Selection

This paper does not explicitly consider the information carrier (genotype) with its readouts (phenotype), nor the environment (bone marrow or thymus or secondary lymph organs in case of the immune cells, intracellular or extracellular space in case of the pathogen). The frequencies of division, the probability of forming new strains during multiplication, and the death rate, all constitute parameters in implicitly dealing with those properties. The discrete time step of the dynamics is given by the duration of each generation. 

We model the process of multiplication and selection by a dynamical stochastic process with the following rules [42]:

(0) Start with one individual *W*_0_^*S*^(1) = 1 (probability 1 of finding one individual at the end of generation *n* = 0). Increase generation number from *n* = 0 to *n* = 1.
(i) Evaluate the probability distribution *W*_*n*_^*M*^(*ν*) of finding 0 ≤ *ν* ≤ *N*_max⁡_ individuals after multiplication phase *M* of generation *n*. The number of individuals reaches the cut-off value *N*_max⁡_ in the case of limited nutrition supply.
(ii) Evaluate the probability distribution *W*_*n*_^*S*^(*N*) of finding 0 ≤ *N* ≤ *N*_max⁡_ individuals after selection phase *S* of generation *n*.
(iii) Increase generation number from *n* to *n* + 1 and continue with (i) accordingly.

### 2.3. Multiplication without Mutation

**Figure 2:**
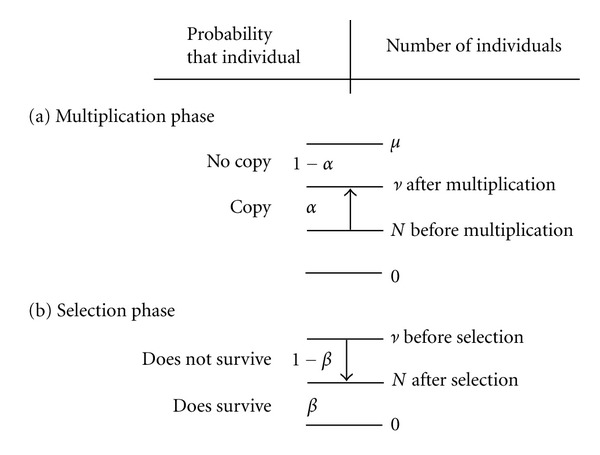
Sketch of how convolution of a binomial distribution is applied to probability distribution. (a) multiplication phase *M*, (3). (b) Selection phase *S*, (4). (*μ* − *N*) Maximal possible number of copies. (*ν* − *N*) Number of copies.

The probability distribution to find 0 ≤ *ν* ≤ *N*_max⁡_ individuals after multiplication phase *M* of the *n*th generation is given by the sum over all possible paths of the conditional probabilities $\left(\begin{array}{c} \mu -N\\ \nu -N \end{array}\right)\alpha^{\left(\nu -N\right)}{\left(1-\alpha \right)}^{\left(\mu -\nu \right)}$ leading to that state (*ν* individuals) given the state (*N* individuals) at the end of the selection phase of the (*n* − 1)th generation times the probability *W*_*n*−1_^*S*^(*N*) of that state, that is, the convolution of a binomial distribution (Figure 2(a))

(3) $$\begin{array}{c} W_{n}^{M}\left(\nu \right)=\sum_{N=\eta }^{\nu }\left(\begin{array}{c} \mu -N\\ \nu -N \end{array}\right)\alpha^{\left(\nu -N\right)}{\left(1-\alpha \right)}^{\left(\mu -\nu \right)}\cdot W_{n-1}^{S}\left(N\right), \end{array}$$

where *α* is the probability of one copy, and 1 − *α* is the probability of no copy. The binomial coefficient counts without regard to order the number of ways of choosing *ν* − *N* copies from *μ* − *N* maximal possible copies, where *ν* is the total number of individuals after the multiplication process, and *μ* = *Min*⁡(*ρ* · *N*, *N*_max⁡_) is the total number of maximal possible individuals after the multiplication process (multiplication factor *ρ*) considering the cut-off condition when the limit of nutrition supply is reached, and *η* = Ceiling(*ν*/*ρ*). *W*_*n*−1_^*S*^(*N*) is the probability of finding the population of *N* individuals before the multiplication process (which is the same as after the selection process of (*n* − 1)th generation). *W*_*n*_^*M*^(*ν*) is a probability distribution with ∑_*ν*=0_^*N*_max⁡_^ *W*_*n*_^*M*^(*ν*) = 1.

The probability distribution to find 0 ≤ *N* ≤ *N*_max⁡_ individuals after the selection phase *S* of the *n*th generation is again given by the sum over all possible paths of the conditional probabilities $\left(\begin{array}{c} \nu \\ N \end{array}\right)\beta^{N}{\left(1-\beta \right)}^{\left(\nu -N\right)}$ leading to that state (*N* individuals), given the state (*ν* individuals) at the end of the multiplication phase of the *n*th generation times the probability *W*_*n*_^*M*^(*ν*) of that state, that is, the convolution of a binomial distribution (Figure 2(b))

(4) $$\begin{array}{c} W_{n}^{S}\left(N\right)=\sum_{\nu =0}^{N_{\max}}\left(\begin{array}{c} \nu \\ N \end{array}\right)\beta^{N}{\left(1-\beta \right)}^{\left(\nu -N\right)}\cdot W_{n}^{M}\left(\nu \right), \end{array}$$

where *β* is the probability that an individual survives, and 1 − *β* is the probability that an individual does not survive. The binomial coefficient counts, without regard to order, the number of ways of choosing *N* surviving individuals from a population of *ν* individuals. The probability of finding this population of *n* individuals before the selection process is *W*_*n*_^*M*^(*ν*). Again, *W*_*n*_^*S*^(*N*) is a probability distribution with ∑_*N*=0_^*N*_max⁡_^ *W*_*n*_^*S*^(*N*) = 1.

**Figure 3:**
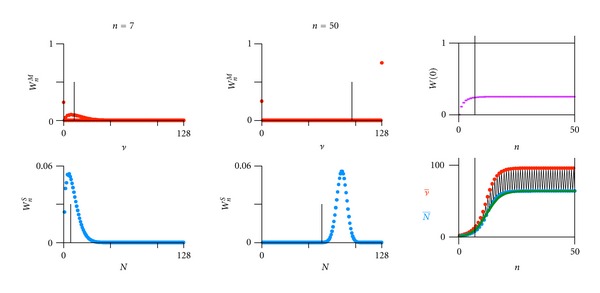
Dynamical stochastic process of multiplication and selection. Discrete limit, one initial ancestor. The two cardinal examples (a) *n* = 7 (*r* ^*n*^-regime, *r* = *ρβ* = 4/3, ln⁡(*r*) = 0.288) and (b) *n* = 50 (*Z*_*∞*_-regime). Upper left: probability distribution *W*_*n*_^*M*^(*ν*) of finding 0 ≤ *ν* ≤ *N*_max⁡_ individuals after the multiplication phase of the *n*th generation (3). Lower left probability distribution *W*_*n*_^*S*^(*N*) of finding 0 ≤ *N* ≤ *N*_max⁡_ individuals after the selection phase of the *n*th generation (4). Maximal total number *N*_max⁡_ (cut-off value due to limited nutrition supply). Upper right: probability of extinction *W*(0) along generation *n*. Lower right: average number of individuals $\overset{-}{\nu }=\sum_{\nu =0}^{N_{\max}}W_{n}^{M}\left(\nu \right)\cdot \nu$ and $\overset{-}{N}=\sum_{N=0}^{N_{\max}}W_{n}^{S}\left(N\right)\cdot N$ along generation *n*. Parameters: *N*_max⁡_ = 128, multiplication factor *ρ* = 2, and copy probability *α* = 1 (all individuals that are present before multiplication copy once), surviving probability *β* = 2/3. Deterministic model (green in lower right, equations (7) and (8)): *K* = 64, *N*_0_ = 1, and *R* = 0.344.

**Figure 4:**
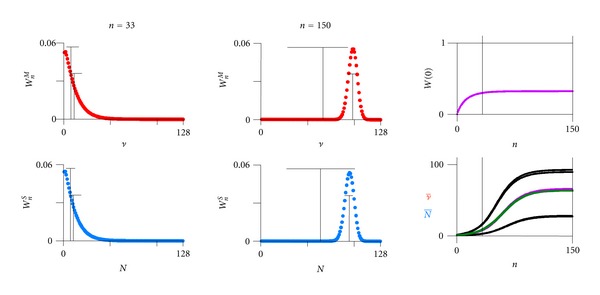
Dynamical stochastic process of multiplication and selection. Continuous limit, one initial ancestor. Two cardinal examples (a) *n* = 33 and (b) *n* = 150. Upper left: probability distribution *W*_*n*_^*M*^(*ν*) of finding *ν* individuals after multiplication phase *M* of generation *n* (3). Lower left: probability distribution *W*_*n*_^*S*^(*N*) of finding *N* individuals after selection phase *S* of generation *n* (4). Average and standard deviation indicated (correct values only by taking the probability of extinction *W*(0) into account). Upper right: probability of extinction *W*(0) along generation *n*. Lower right: number of individuals $\overset{-}{\nu }$ and $\overset{-}{N}$ with its standard deviation along generation *n* (least square fit, *R* = 0.0694, see (7) and (8)). Parameters: Maximal total number *N*_max⁡_ = 128, multiplication factor *ρ* = 2, and copy probability *α* = 0.1 (only 10% of individuals that are present before multiplication copy once), surviving probability *β* = 0.9664.

We assume that one initial ancestor appears in the beginning of the first generation (which is the same as after the selection process of generation *n* = 0) thus the probability *W*_0_^*S*^(1) = 1 (root of iterative process). The numerical evaluations are given in Figure 3 (discrete limit) and Figure 4 (continuous limit). How the model responds to variations in key parameters is presented in [42]. One can insert (3) into (4) (renaming function and index, see (5)) and construct the probability generating function (PGF) with the dummy variable *s*, see (6)

(5) $$\begin{array}{c} P_{N}\left(j\right)=\sum_{\nu =j}^{\mu }\left(\begin{array}{c} \mu -N\\ \nu -N \end{array}\right)\alpha^{\left(\nu -N\right)}{\left(1-\alpha \right)}^{\left(\mu -\nu \right)}\cdot \left(\begin{array}{c} \nu \\ j \end{array}\right)\beta^{j}{\left(1-\beta \right)}^{\left(\nu -j\right)}, \end{array}$$

(6) $$\begin{array}{c} G_{N}\left(s\right)=\sum_{j=0}^{N_{\max}}P_{N}\left(j\right)\cdot s^{j}={\left(1-\beta +\beta s\right)}^{N}{\left(1-\alpha \beta +\alpha \beta s\right)}^{N}. \end{array}$$

A step-by-step Darwinian process alternating between a multiplication and a selection phase per generation with a constant generation time (typically a fraction of an hour to days) and nonoverlapping generations can be contrived in the two following ways:

- By the discrete limit with great oscillations in average numbers of individuals $\overset{-}{\nu }$ and $\overset{-}{N}$ (Figure 3), where each individual is copied (copy probability *α* = 1), and where the selection is intermediate (selection probability to survive *β* = 2/3). This would imply an orchestration of phases by an environmental pacemaker (day and night in case of origin of life [42–46]).
- By the continuous limit (Figure 4), where the probability of an individual to copy is sufficiently small (*α* = 0.1), and where the probability to be selected to survive is close to one (*β* = 0.9664). This implies individual pacemakers and a smother course of the average numbers of individuals $\overset{-}{\nu }$ and $\overset{-}{N}$.

Taking average values of the probability distributions, one compares the stochastic model with the deterministic model given by the differential equation [47] describing a birth-death process (number of individuals *N*, *R* = birth  rate − death  rate and saturation *K*, where *dN*/*dt* = 0) and its analytic solution (initial value *N*_0_ of *N*)

(7) $$\begin{array}{c} \frac{dN}{dt}=RN-\frac{R}{K}N^{2}, \end{array}$$

(8) $$\begin{array}{c} N\left(t\right)=e^{Rt}\frac{N_{0}K}{\left(K-N_{0}+N_{0}e^{Rt}\right)}. \end{array}$$

Note: the probability distribution *W*(*N*) and the probability of extinction *W*(0) are not represented within deterministic models.

### 2.4. Multiplication with Copying Errors Leading to One Mutant

**Figure 5:**
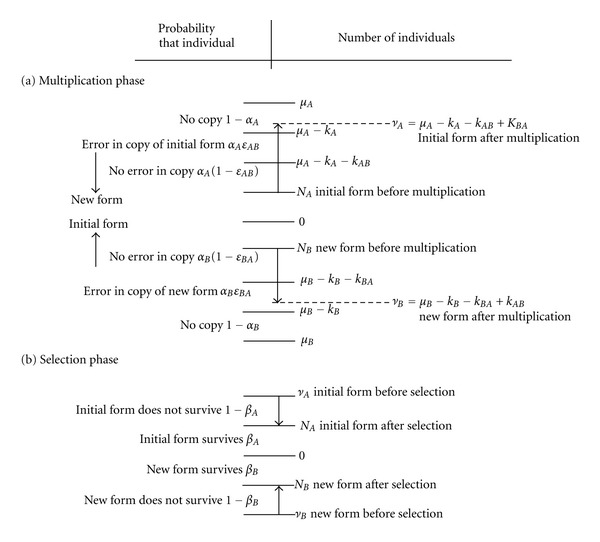
Sketch for (9), (10), and (12) of how convolution of a binomial distribution is applied to probability distribution. (a) Multiplication phase *M*, (9), (10): *μ*_*A*_ − *N*_*A*_ − *k*_*A*_ total copies of initial form *A* (and *μ*_*B*_ − *N*_*B*_ − *k*_*B*_ total copies of mutant *B*); *μ*_*A*_ − *N*_*A*_ maximal possible copies according to the multiplication factors *ρ*_*A*_ of initial form *A* (and *μ*_*B*_ − *N*_*B*_ maximal possible copies according to the multiplication factors *ρ*_*B*_ of mutant *B*); *k*_*AB*_ (and *k*_*BA*_) number of copies transforming from initial form *A* to mutant *B* (and vice versa resp.). (b) Selection phase *S*, (12).

The probability distribution to find *ν*_*A*_ individuals of the initial form *A* and *ν*_*B*_ individuals of the mutant *B*  (0 ≤ *ν*_*A*_ + *ν*_*B*_ = *ν* ≤ *N*_max⁡_) after the multiplication phase *M* of the *n*th generation is given by the convolution (Figure 5(a))

(9) WnM(νA,νB)  =∑NA=0Nmax⁡ ∑NB=0Nmax⁡−NA ∑kA=0μA−NA ∑kAB=0μA−kA−NAWn−1S(NA,NB)   ·(μA−NAkA)αA(μA−kA−NA)(1−αA)kA   ·(μA−kA−NAkAB)εABkAB(1−εAB)(μA−kA−kAB−NA)   ·(μB−NBkB)αB(μB−kB−NB)(1−αB)kB   ·(μB−kB−NBkBA)εBAkBA(1−εBA)(μB−kB−kBA−NB),

where the two remaining indices are given by

(10) $$\begin{array}{c} k_{B}=\mu_{A}+\mu_{B}-\nu_{A}-\nu_{B}-k_{A},\\ k_{BA}=\nu_{A}+k_{AB}+k_{A}-\mu_{A}. \end{array}$$

**Figure 6:**
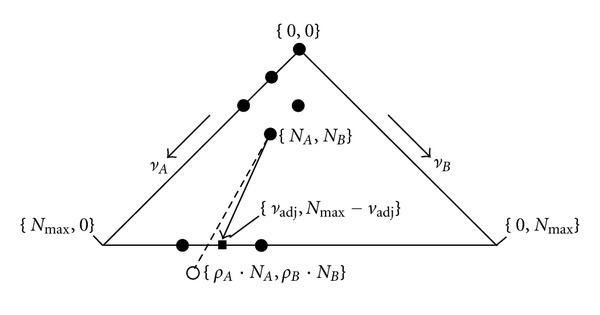
Sketch to construct the cut-off condition in the case of initial form *A* and one kind of mutant *B*. If, by multiplication of {*N*_*A*_, *N*_*B*_} individuals (black disk •) with factors *ρ*_*A*_ and *ρ*_*B*_ respectively, the total number *ρ*_*A*_ · *N*_*A*_ + *ρ*_*B*_ · *N*_*B*_ > *N*_max⁡_ would be beyond the limit of supply *N*_max⁡_, (circle ∘), the correct cut-off point {*ν*_adj_, *N*_max⁡_ − *ν*_adj_} (black square ■) is the most adjacent to the intersection. Note: partition and take average for more than one such point. The probability distributions and cut-off conditions in the cases of more than one kind of mutant are accordingly.

Each individual *A* (and *B*) replicates by a copy probability *α*_*A*_ (and *α*_*B*_, resp.). *ε*_*AB*_ (and *ε*_*BA*_) are the probabilities that an error in copying the initial form *A* occurs, and the new form *B* emerges (and vice versa). The second binomial coefficient (and the fourth binomial coefficient) counts without regard to order the number of ways of choosing *k*_*AB*_ (and *k*_*BA*_, resp.) error-containing copies of the initial form *A* giving the new form *B* (and vice versa) from a collection of *μ*_*A*_ − *k*_*A*_ − *N*_*A*_  (and  *μ*_*B*_ − *k*_*B*_ − *N*_*B*_) total copies of the initial form *A* (and of the new form *B*, resp.). The first binomial coefficient (and the third binomial coefficient) counts without regard to order the number of ways of choosing *μ*_*A*_ − *k*_*A*_ − *N*_*A*_  (and  *μ*_*B*_ − *k*_*B*_ − *N*_*B*_) total copies of the initial form *A* (of the new form *B*, resp.) from a collection of *μ*_*A*_ − *N*_*A*_  (and  *μ*_*B*_ − *N*_*B*_) maximal possible copies according to the multiplication factors *ρ*_*A*_  (and  *ρ*_*B*_) of the initial form *A* (and of the new form *B*, resp.). The cut-off conditions (where *N*_max⁡_ is for the total numbers of both forms *A* and *B* and *ν*_adj_ is defined in Figure 6) then are

(11) $$\begin{array}{c} \mu_{A}=\rho_{A}\cdot N_{A}\;\mu_{B}=\rho_{B}\cdot N_{B}\;\text{if}\;\rho_{A}\cdot N_{A}+\rho_{B}\cdot N_{B}\leq N_{\max},\\ \mu_{A}=\nu_{\text{adj}}\;\mu_{B}=N_{\max}-\nu_{\text{adj}}\;\text{if}\;\rho_{A}\cdot N_{A}+\rho_{B}\cdot N_{B}>N_{\max}. \end{array}$$

The probability distribution to find *N*_*A*_ individuals of the initial form *A* and *N*_*B*_ individuals of the new form (mutant *B*) (0 ≤ *N*_*A*_ + *N*_*B*_ = *N* ≤ *N*_max⁡_) after the selection phase *S* of the *n*th generation is given by the convolution (Figure 5(b))

**Figure 7:**
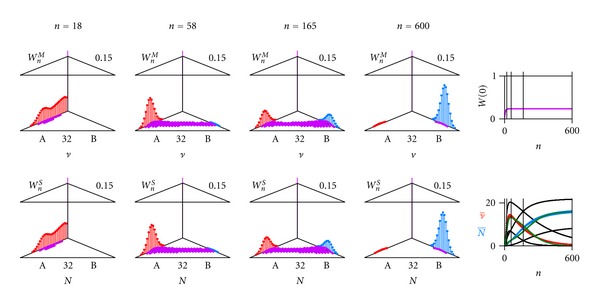
Dynamical stochastic process for initial form *A* and mutant *B*. One initial ancestor of form *A*. Three cardinal examples (a) *n* = 48, (b) *n* = 164, and (c) *n* = 600. Upper left (triangle graph for 0 and scales 0.15 for *W* and 32 for *n*): probability distribution *W*_*n*_^*M*^(*ν*_*A*_, *ν*_*B*_) of finding *ν*_*A*_ individuals of initial form *A* (red) and *ν*_*B*_ individuals of mutant *B* (blue) after multiplication phase *M* of generation *n* (9), (10). Lower left (triangle graph): probability distribution *W*_*n*_^*S*^(*N*_*A*_, *N*_*B*_) of finding *N*_*A*_ individuals of initial form *A* (red) and *N*_*B*_ individuals of mutant *B* (blue) after selection phase *S* of generation *n* (12). Upper right: total extinction probability *W*(0) of both initial form (*A*) and mutant (*B*) together (violet) along generation *n*. Lower right: average number of individuals $\overset{-}{\nu }$ and $\overset{-}{N}$ of initial form *A* (red) and mutant *B* (blue) with their standard deviations (black) along generation *n*. Parameters: maximal total number *N*_max⁡_ = 32, copy probability *α*_*A*_ = 0.1 and *α*_*B*_ = 0.1, multiplication factor *ρ*_*A*_ = 3 of initial form *A*, multiplication factor *ρ*_*B*_ = 6 of mutant *B*, mutation probability *ε*_*AB*_ = 0.01 from initial form *A* to mutant *B*, mutation probability *ε*_*BA*_ = 0.001 from mutant *B* to initial form *A*, surviving probability *β*_*A*_ = 0.95 of initial form *A*, surviving probability *β*_*B*_ = 0.95 of mutant *B*. Deterministic model (green in lower right, (13)): *R*_*a*_ = 0.128, *R*_*b*_ = 0.14, and *K* = 16.

(12) WnS(NA,NB)=∑νA=NANmax⁡ ∑νB=NBNmax⁡−νAWnM(νA,νB)·(νANA)βANA(1−βA)(νA−NA)·(νBNB)βBNB(1−βB)(νB−NB),

where *β*_*A*_ (and *β*_*B*_) are the probabilities that one individual of the initial form *A* (and mutant *B*, resp.) survives.  Figure 7 shows the dynamics resulting from evaluation by computer. 

Taking average values of the probability distributions, one compares the stochastic model with the deterministic model given by the two coupled differential equations [48] being the extension of a birth-and-death process described in (7) for two entities transforming one entity into the other (number of individuals *N*_*A*_ and *N*_*B*_, *R* = birth  rate − death  rate parameters *R*_*a*_ and *R*_*b*_, and saturation *K*) as follows:

(13) $$\begin{array}{c} \frac{dN_{A}}{dt}=R_{a}\cdot N_{A}-\frac{R_{a}}{K}N_{A}^{2}-\frac{R_{b}}{K}N_{A}N_{B},\\ \frac{dN_{B}}{dt}=R_{b}\cdot N_{B}-\frac{R_{b}}{K}N_{B}^{2}-\frac{R_{a}}{K}N_{B}N_{A}. \end{array}$$

## 3. Applying the Methods

### 3.1. Modeling the Immune Response to a Pathogen by Coupling Two Darwinian Entities

Let us look first at the conceptual fundamentals of our model: to get the general idea, one applies Occam's razor (or lex parsimoniae translating to law of succinctness) onto the complex immunological system described above and then one provides a minimal representation of the immune response. In the following we consider:

- the host as being unstructured by not considering its multicompartmentness (i.e., not considering that the entrance site of the pathogen is spatially apart from the corresponding secondary lymph organ, where part of the immune-system response takes place);
- the invading pathogen (*P*) taking into account its variable antigens but not distinguishing between endogenous or exogenous paths (i.e., not considering that the pathogen thrives within a cell of the host or within the interstitial space);
- the immune system with the immune effector (*E*) taking into account an immune memory (*M*), but not distinguishing between T or B lymphocytes;
- the step-by-step Darwinian process as fundamental to both entities (pathogen as well as to the immune effector and its memory state), which are specifically coupled;
- the stochastic representation of a Darwinian entity as a sufficiently good starting point to solve the proposed problem.

Stochastic models offer the benefit of handling the dynamics of whole distributions with their mean and standard deviation as deduction, whereas deterministic models deal with quantities that arise as large population rescaling. 

**Figure 8:**
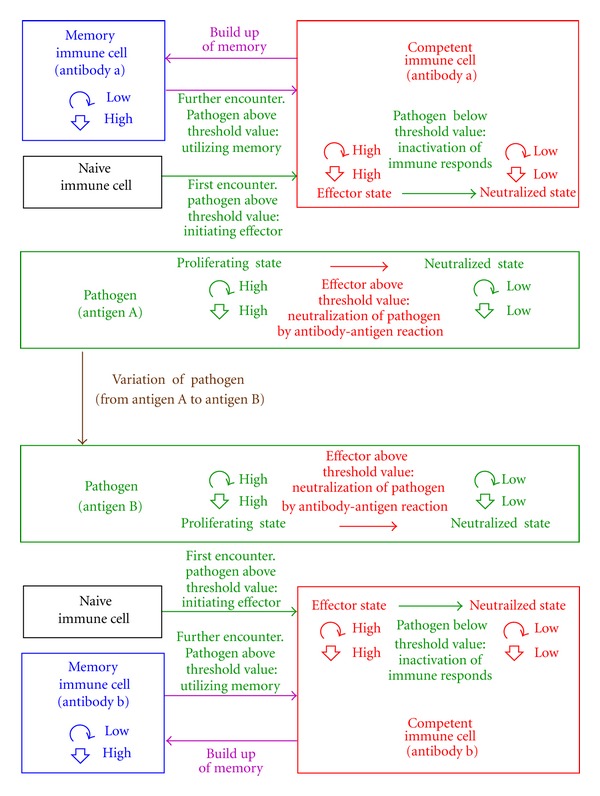
Scheme of the immune system response (immune effectors *E*_*a*_ producing antigen-receptor a and immune-memory cells *M*_*a*_) to an invading pathogen (pathogen *P*_*A*_ carrying antigen *A*). Two-state model: immune effector is turned on (from neutralized state to effector state) when average number of pathogens go above threshold *T*_*P*_; pathogen is turned off (from proliferating to neutralized state) when average immune response goes above threshold *T*_*E*_; immune effector is turned off (from effector state to neutralized state) when average number of pathogens go below threshold. First infection by the proliferating pathogen *P*_*A*_ (high multiplication factor, high selection probability to survive) initiates the immune response of the host: an immune cell with the recipe for antigen-receptor “*a*” is singled out from the reservoir of naive immune cells to form an immune effector *E*_*a*_ which proliferates (high-multiplication factor, high-selection probability). Above *a* threshold titer of antigen-receptor *a*, the pathogen is neutralized *P*_*A*_ (low multiplication factor, low selection probability). The immune effectors *E*_*a*_ are transformed into immune-memory cells *M*_*a*_ which do not produce antigen-receptor a but carry its recipe (low-multiplication factor, high-selection probability). During any further infections by the pathogen *P*_*A*_, the immune-memory cells with the recipe for antigen-receptor *a* are formed back into immune effectors producing antigen-receptor *a*. Pathogen *P*_*A*_ carrying antigen *A* can transform to pathogen *P*_*B*_ carrying antigen *B*. A new immune respond has to be launched with immune effectors *E*_*b*_ producing antigen-receptor *b* and immune-memory cells *M*_*b*_.

We propose a dynamical model of two interlocked Darwinian entities, the pathogen *P* on the one hand, and the immune system on the other hand consisting of the immune effector *E* and the immune memory *M* (Figure 8). The coupling is such that at each time step the parameters for the pathogen system are dependent on the current state of the immune system and the parameters for the immune system are dependent on the current state of the pathogen system. As in any control system (such as body temperature of endotherms or glucose concentration in blood) there are two states: (i) the measured variable goes below a threshold or “lower set point,” then the actuator is turned on and subsequently the measured value increases, and (ii) the measured variable goes above a threshold or “upper set point,” then the actuator is turned off and subsequently the measured value decreases. Within stochastic fluctuations such systems are intrinsic periodic around a steady state.

### 3.2. Modeling the Immune Response to One Pathogen

The multiplication phase of pathogen *P* (with antigen *A*) is described by (see (3) from Section, now with index *P*)

(14) WnM(νP) =∑NP=ην(μP−NPνP−NP)αP(νP−NP)(1−αP)(μP−νP)·Wn−1S(NP).

The selection phase of pathogen *P* is described by (see (4) from Section, now with index *P*)

(15) WnS(NP) =∑ν=NPNmax⁡(νPNP)βPNP(1−βP)(νP−NP)·WnM(νP).

The multiplication phase of immune effector *E* (producing antibody *a*) and memory *M* is described by (see (9) and (10) from Section 2.4, now with indices *E* and *M*)

(16) WnM(νE,νM)=∑NE=0Nmax⁡ ∑NM=0Nmax⁡−NE ∑kE=0μE−NE ∑kEM=0μE−kE−NEWn−1S(NE,NM) ·(μE−NEkE)αE(μE−kE−NE)(1−αE)kE ·(μE−kE−NEkEM)εEMkEM(1−εEM)(μE−kE−kEM−NE) ·(μM−NMkM)αM(μM−kM−NM)(1−αM)kM ·(μM−kM−NMkME)εMEkME(1−εME)(μM−kM−kME−NM),

where the two remaining indices are given by

(17) $$\begin{array}{c} k_{M}=\mu_{E}+\mu_{M}-\nu_{E}-\nu_{M}-k_{E},\\ k_{ME}=\nu_{E}+k_{EM}+k_{E}-\mu_{E}. \end{array}$$

The cut-off conditions are

(18) μE=ρE·NEμM=ρM·NM if  ρE·NE+ρM·NM≤Nmax⁡,μE=νadjμM=Nmax⁡−νadj if  ρE·NE+ρM·NM>Nmax⁡,μP=ρP·NP if  ρP·NP≤Nmax⁡,μP=Nmax⁡ if  ρP·NP>Nmax⁡.

The selection phase of immune effector *E* and memory *M* is described by (see (12) from Section 2.4, now with indices *E* and *M*)

(19) WnS(NE,NM)=∑νE=NENmax⁡∑νM=NMNmax⁡−νEWnM(νE,νM)·(νENE)βENE(1−βE)(νE−NE)·(νMNM)βMNM(1−βM)(νM−NM).

Each individual has a probability *β*, that is, $\beta_{P}\left(\overset{-}{E}\right)$, $\beta_{E}\left(\overset{-}{P}\right)$, and $\beta_{M}\left(\overset{-}{P}\right)$, being selected to survive, it replicates by a copy-probability *α* = 0.1, multiplication-factor *ρ*, that is, $\rho_{P}\left(\overset{-}{E}\right)$, $\rho_{E}\left(\overset{-}{P}\right)$, and $\rho_{M}\left(\overset{-}{P}\right)$ with a mutation probability *ε*, that is, $\varepsilon_{EM}\left(\overset{-}{P}\right)$ and $\varepsilon_{ME}\left(\overset{-}{P}\right)$. Thus, the multiplication factor *ρ*, the error probability *ε*, and the surviving probability *β* are parameters in function of averages $\overset{-}{P}=\sum_{N_{P}=0}^{N_{P,\max}}W\left(N_{P}\right)\cdot N_{P}$ and $\overset{-}{E}=\sum_{N_{E},N_{M}=0}^{N_{\max}}W\left(N_{E},N_{M}\right)\cdot N_{E}$ (omitting the indices *S*/*M* and *n* for simplicity) that form the coupling (step functions with threshold values, see captions of Figures 8, 2 and 4). 

**Figure 9:**
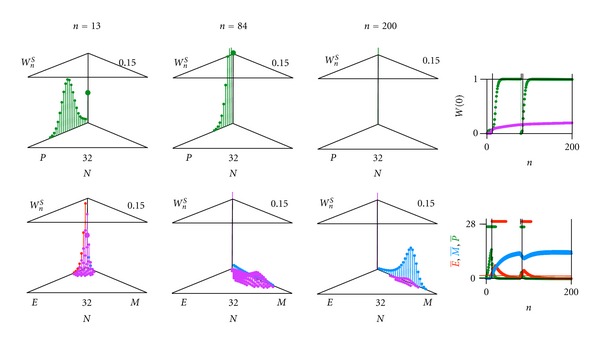
Computer result of a pathogen *P* (with antigen *A*) coupled through averages to an immune system consisting of an effector *E* (producing antigen receptor *a*) and memory *M*. Case where pathogen is eliminated. Glimpses at generation (a) *n* = 13, (b) *n* = 84 and (c) *n* = 200. Upper left (triangle graph for 0 and scales 0.15 for *W* and 32 for *N*): probability *W*_*n*_^*S*^(*N*_*P*_) of finding 0 ≤ *N*_*P*_ ≤ *N*_max⁡_ individuals of the pathogen *P* (green). Lower left (triangle graph): probability *W*_*n*_^*S*^(*N*_*E*_, *N*_*M*_) of finding *N*_*E*_ individuals of the immune effector *E* (red) and *N*_*M*_ individuals of the immune memory *M* (blue), (0 ≤ *N*_*E*_ + *N*_*M*_ ≤ *N*_max⁡_). Upper right: extinction probability *W*(0) as a function of generations *n* of pathogen (green), immune effector, and immune memory (violet). Lower right: average of pathogen $\overset{-}{P}$ (green), of immune effector $\overset{-}{E}$ (red), and of immune memory $\overset{-}{M}$ (blue) as a function of generations *n*. Parameter values: maximal total number *N*_max⁡_ = 32; *α*_*P*_ = 0.1, *α*_*E*_ = 0.1, and *α*_*M*_ = 0.1. For average $\overset{-}{E}<T_{E}=1.5$ (below threshold value, immune effector *E* inactive): *ρ*_*P*_ = 6, *β*_*P*_ = 0.96. For average $\overset{-}{E}>T_{E}=1.5$ (above threshold value, immune effector *E* active): *ρ*_*P*_ = 2, *β*_*P*_ = 0.65. For average $\overset{-}{P}>T_{P}=0.5$ (below threshold value, pathogen *P* not seen by immune system): *ρ*_*E*_ = 2, *ρ*_*M*_ = 2, *ε*_*EM*_ = 0.50, *ε*_*ME*_ = 0.01, *β*_*E*_ = 0.90, and *β*_*M*_ = 0.93. For average $\overset{-}{P}>T_{P}=0.5$ (above threshold value, pathogen *P* seen by immune system): *ρ*_*E*_ = 6, *ρ*_*M*_ = 2, *ε*_*EM*_ = 0.50, *ε*_*ME*_ = 0.50, *β*_*E*_ = 0.95, and *β*_*M*_ = 0.93.

**Figure 10:**
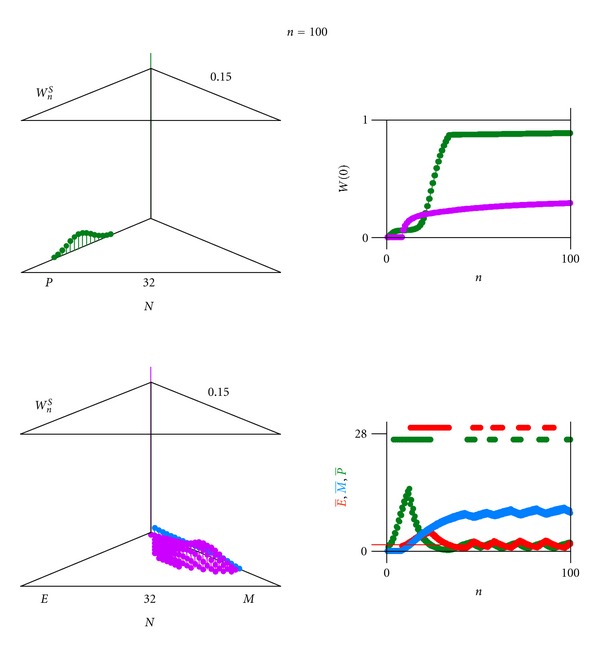
Computer result of a pathogen *P* (with antigen *A*) coupled through averages to an immune system consisting of an effector *E* (producing antigen receptor *a*) and memory *M*. Case where the pathogen is reappearing while the immune system response is low. Glimpse at generation *n* = 100. Parameter values other than Figure 2: For average $\overset{-}{E}>T_{E}=1.5$ (above threshold value, immune effector *E* active): *β*_*P*_ = 0.75. For average $\overset{-}{P}<T_{P}=1.5$ (below threshold value, pathogen *P* not seen by immune system): *β*_*E*_ = 0.85. For average $\overset{-}{P}>T_{P}=1.5$ (above threshold value, pathogen *P* seen by immune system): *β*_*E*_ = 0.90.

In Figure 9 the case is shown, where the pathogen is eliminated (probability of extinction *W*(0) = 1.0 after initial infection and after reinfection). In Figure 10, an oscillatory chronic case is shown, where after an apparent conquest and the subsequent relaxation of the immune reaction, the pathogen is flaring up again (probability of extinction *W*(0) persists below 1.0).

### 3.3. Modeling the Immune Response to a Variable Pathogen

**Figure 11:**
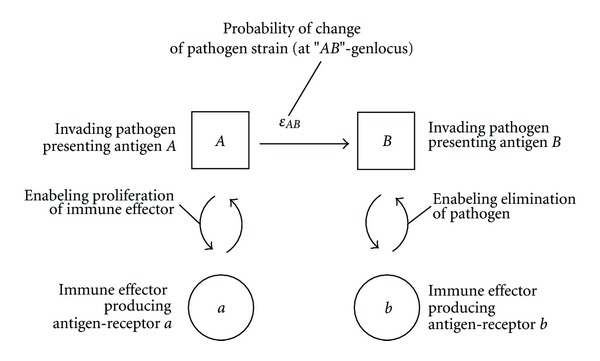
Immune response to a variable pathogen (pathogen strains each with different antigens which are presented to the immune system). Two alleles at a *AB*-genlocus of the pathogen expressing antigen *A* or *B*: the immune effectors respond specifically (lock and key principle) by proliferation and producing antigen-receptor (antibody) *a* or *b*.

We consider a simple case (Figure 11) of a pathogen with two alleles at an *A*-to-*B* genlocus (one epitope): the pathogen *P*_*A*_ (or pathogen *P*_*B*_, resp.) expressing antigen *A* (or antigen *B*). The multiplication phase of pathogens *P*_*A*_ and *P*_*B*_ is described by ((9), (10) from Section 2.4, now with indices *P*_*A*_ and *P*_*B*_)

(20) WnM(νPA,νPB) =∑NPA=0Nmax⁡ ∑NPB=0Nmax⁡−NPA ∑kPA=0μPA−NPA ∑kPAPB=0μPA−kPA−NPAWn−1S(NPA,NPB)   .(μPA−NPAkPA)αPA(μPA−kPA−NPA)(1−αPA)kPA   .(μPA−kPA−NPAkPAPB)εPAPBkPAPB(1−εPAPB)(μPA−kPA−kPAPB−NPA)   .(μPB−NPBkPB)αPB(μPB−kPB−NPB)(1−αPB)kPB   .(μPB−kPB−NPBkPBPA)εPBPAkPBPA(1−εPBPA)(μPB−kPB−kPBPA−NPB),

where the two remaining indices are given by

(21) $$\begin{array}{c} k_{P_{B}}=\mu_{P_{A}}+\mu_{P_{B}}-\nu_{P_{A}}-\nu_{P_{B}}-k_{P_{A}},\\ k_{P_{B}P_{A}}=\nu_{P_{A}}+k_{P_{A}P_{B}}+k_{P_{A}}-\mu_{P_{A}}. \end{array}$$

The selection phase of pathogens *P*_*A*_ and *P*_*B*_ is described by ((12) from section 2.4, now with indices *P*_*A*_ and *P*_*B*_)

(22) WnS(NPA,NPB)=∑νPA=NPANmax⁡∑νPB=NPBNmax⁡−νPAWnM(νPA,νPB).(νPANPA)βPANPA(1−βPA)(νPA−NPA).(νPBNPB)βPBNPB(1−βPB)(νPB−NPB).

The immune effector *E*_*a*_ (or immune effector *E*_*b*_) responding specifically, thus producing antigen-receptor a (or antigen-receptor *b*) which recognizes the antigen *A* (or antigen *B*) and eliminates pathogen *P*_*A*_ (or pathogen *P*_*B*_, resp.). While the antigen “*A*” is the “lock” and the antigen-receptor “*a*” is the corresponding “key” (or the antigen *B* is the lock and the antigen-receptor *b* is another, but corresponding key.

**Figure 12:**
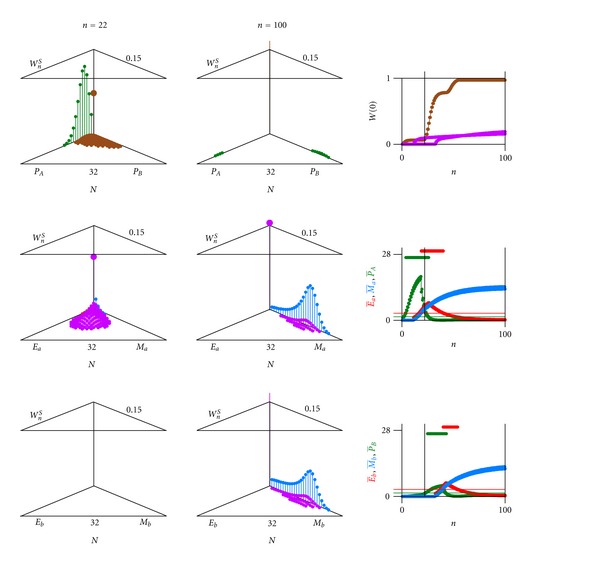
Computer result of a varying pathogen (*P*_*A*_ with antigen *A* changing into *P*_*B*_ with antigen *B*) coupled through averages to an immune system against antigen *A* consisting of an effector *E*_*a*_ and memory *M*_*a*_ and against antigen *B* consisting of an effector *E*_*b*_ and memory Mb. Partial change of pathogen *P*_*A*_ to pathogen *P*_*B*_ escaping immune effector *E*_*a*_ and with delayed immune response of effector *E*_*b*_. Glimpses at generation (a) *n* = 22, (b) *n* = 100. Upper left (triangle graph for 0 and scales 0.15 for *W* and 32 for *N*): probability *W*_*n*_^*S*^(*N*_*P*_*A*__, *N*_*P*_*B*__) of finding *N*_*P*_*A*__ individuals of the pathogen *P*_*A*_ with antigen *A* and of finding *N*_*P*_*B*__ individuals of the pathogen *P*_*B*_ with antigen *B* (green) (0 ≤ *N*_*P*_*A*__ + *N*_*P*_*B*__ ≤ *N*_max⁡_). Middle left (triangle graph): probability *W*_*n*_^*S*^(*N*_*E*_*a*__, *N*_*M*_*a*__) of finding *N*_*E*_*a*__ individuals of the immune effector (red) and *N*_*M*_*a*__ individuals of immune memory (blue) against antigen *A* (0 ≤ *N*_*E*_*a*__ + *N*_*M*_*a*__ ≤ *N*_max⁡_). Lower left (triangle graph): probability *W*_*n*_^*S*^(*N*_*E*_*b*__, *N*_*M*_*b*__) of finding *N*_*E*_*b*__ individuals of the immune effector (red) and *N*_*M*_*b*__ individuals of immune memory (blue) against antigen *B* (0 ≤ *N*_*E*_*b*__ + *N*_*M*_*b*__ ≤ *N*_max⁡_). Upper right: extinction probability *W*(0) as a function of generations *n* of pathogen (green), immune effector & memory against antigen *A* and immune effector & memory against antigen *B* (both violet). Middle and lower right: average of pathogen $\overset{-}{P}$ (green), of immune effector $\overset{-}{E}$ (red) and of immune memory $\overset{-}{M}$ (blue) against antigen *A* (middle right) and against antigen *B* (lower right) as a function of generations *n*. Parameter values others than Figure 2: average *T*_*P*_*A*__ = 1.5 and average *T*_*P*_*B*__ = 1.5, respectively (threshold value to switch immune system); *ε*_*P*_*A*_*P*_*B*__ = 0.01, *ε*_*P*_*B*_*P*_*A*__ = 0.001 (pathogen variability).

In Figure 12 we show a computer result of a varying pathogen (*P*_*A*_ with antigen *A* changes into *P*_*B*_ with antigen *B* with a certain probability *ε*_*P*_*A*_*P*_*B*__, *ε*_*P*_*B*_*P*_*A*__ vice versa, see equations (20)–(22) with indices *P*_*A*_ und *P*_*B*_ describing pathogens *A* and *B*) coupled through average values to an immune system against antigen *A* (and against antigen *B*) consisting of an effector *E*_*a*_ and memory *M*_*a*_ (and an effector *E*_*b*_ and memory *M*_*b*_). There is no “*a* to *b*” or “*b* to *a*”—transition within the immune system. The pathogen expressing antigen *A* is nearly eradicated, but the mutant pathogen strain-expressing antigen *B* has escaped the immune attack (probability distribution upper left of Figure 12). As an outcome, one can see that the thriving of the pathogen within the host is prolonged.

## 4. Maturation of T-Lymphocytes

**Figure 13:**
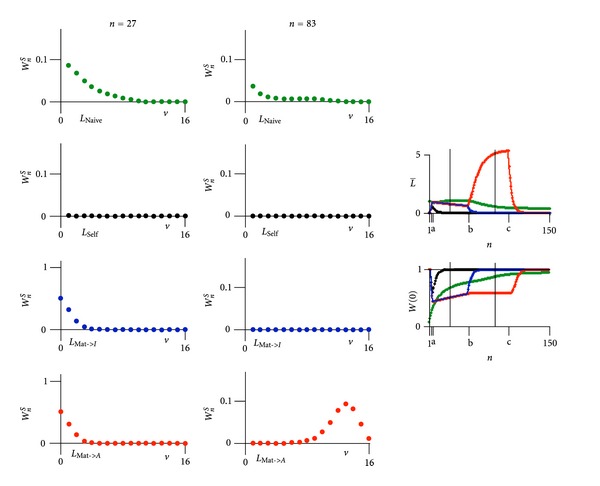
Computer result of the maturation process of T-lymphocytes. First a naïve T-lymphocyte (*L*_Naive_, green) in bone marrow or thymus undergoes T-cell receptor rearrangement (*β*-selection). T-cells with high affinity to self-peptides MHC (*L*_Self_, black) are eliminated (negative selection), whereas T-cells with T cell receptors that are able to bind self-peptides MHC molecules with at least a weak affinity (*L*_Mat->*I*_, blue and *L*_Mat->*A*_, red) survive (positive selection) and circulate in the peripheral lymphatic system. The matured T-lymphocyte, recognizing the antigen by high affinity to the antigen-loaded MHC (*L*_Mat->*A*_, red), transforms into an effector cell and proliferates. Glimpses at generation at *n* = 27 and *n* = 83. Left: probabilities *W*_*n*_^*S*^(*N*) of finding *N* individuals of T lymphocytes (0 ≤ *N* ≤ *N*_max⁡_). Upper right: extinction probability *W*(0) as a function of generations *n*. Lower right: average of T lymphocytes $\overset{-}{L}$ as a function of generations n. Parameter values and their change during the dynamics (a) *n* < 50 (b) 50 ≤ *n* < 100 (c) 100 ≤ *n*: maximal total number *N*_max⁡_ = 16; *α*_*L*_Naive__ = 0.1, *α*_*L*_Self__ = 0.1, *α*_*L*_Mat->*I*__ = 0.1, *α*_*L*_Mat->*A*__ = 0.1, *ρ*_*L*_Naive__ = 2, *ρ*_*L*_Self__ = 1, *ρ*_*L*_Mat->*I*__ = 1, *ρ*_*L*_Mat->A__ = 1/4/1, *β*_*L*_Naive__ = 0.95, *β*_*L*_Self__ = 0.75, *β*_*L*_Mat->*I*__ = 0.99/0.75/0.75, *β*_*L*_Mat->*A*__ = 0.99/0.98/0.75, *ε*_*L*_Naive_*L*_Self__ = 0.7, *ε*_*L*_Naive_*L*_Mat->*I*__ = 0.7, *ε*_*L*_Naive_*L*_Mat->*A*__ = 0.7.

As mentioned in Section 1.2, the T-lymphocyte comes in four different forms: a naïve T-lymphocyte in bone marrow or thymus undergoes T-cell receptor rearrangement (*β*-selection), where T-cells with high affinity to self-peptides MHC are eliminated (negative selection), and T-cells with T cell receptors that are able to bind MHC molecules with at least a weak affinity survive in the peripheral lymphatic system (positive selection). The matured T-lymphocyte recognizing the antigen by high affinity to the antigen loaded MHC transforms into an effector cell and proliferates. We consider in Figure 13 the dynamics of a probability function with four variables describing naïve lymphocyte *L*_Naive_, lymphocyte *L*_Self_ with strong affinity to self-peptides, matured lymphocyte *L*_Mat−>*I*_ with weak affinity to foreign-peptides, this lymphocyte gets inactivated, matured lymphocyte *L*_Mat−>*A*_ with strong affinity to foreign-peptides, this lymphocyte gets activated (Mat = matured, *I* = inactivated, *A* = activated). The multiplication phase is described by

(23) WnM(νLNaive,νLSelf,νLMat->I,νLMat->A)=∑NLNaive=0Nmax⁡ ∑NLSelf=0Nmax⁡−NLNaive ∑NLMat->I=0Nmax⁡−NLNaive−NLSelf ∑NLMat->A=0Nmax⁡−NLNaive−NLSelf−NLMat->I Wn−1S(NLNaive,NLSelf,NLMat->I,NLMat->A)·(μLNaive−NLNaivekLNaive)αLNaive(μLNaive−kLNaive−NLNaive)(1−αLNaive)kLNaive·(μLSelf−NLSelfkLSelf)αLSelf(μLSelf−kLSelf−NLSelf)(1−αLSelf)kLSelf·(μLMat->I−NLMat->IkLMat->I)αLMat->I(μLMat->I−kLMat->I−NLMat->I)·(1−αLMat->I)kLMat->I·(μLMat->A−NLMat->AkLMat->A)αLMat->A(μLMat->A−kLMat->A−NLMat->A)·(1−αLMat->A)kLMat->A,

where the remaining indices are given by

(24) $$\begin{array}{c} k_{L_{\text{Naive}}}=\mu_{L_{\text{Naive}}}-\nu_{L_{\text{Naive}}},\\ k_{L_{\text{Self}}}=\mu_{L_{\text{Self}}}-\nu_{L_{\text{Self}}},\\ k_{L_{\text{Mat-}>I}}=\mu_{L_{\text{Mat-}>I}}-\nu_{L_{\text{Mat-}>I}},\\ k_{L_{\text{Mat-}>A}}=\mu_{L_{\text{Mat-}>A}}-\nu_{L_{\text{Mat-}>A}}. \end{array}$$

For the phase (a) in Figure 13, the multiplication phase is described by inserting formula (25) into formula (23)

(25) ∑kLSelf=0μLSelf−NLSelf  ∑kLMat−>I=0μLMat−>I−NLMat−>I 16∑Permutation(LSelf,LMat−>I,LMat−>A)[(μLNaive−NLNaivekLNaiveLSelf)·εLNaiveLSelfkLNaiveLSelf(1−εLNaiveLSelf)(μLNaive−NLNaive−kLNaiveLSelf)·(μLNaive−NLNaive−kLNaiveLSelfkLNaiveLMat−>I)·εLNaiveLMat−>IkLNaiveLMat−>I·(1−εLNaiveLMat−>I)(μLNaive−NLNaive−kLNaiveLSelf−kLNaiveLMat−>I)·(μLNaive−NLNaive−kLNaiveLSelf−kLNaiveLMat−>IkLNaiveLMat−>A)·εLNaiveLMat→AkLNaiveLMat→A·(1−εLNaiveLMat−>A)(μLNaive−NLNaive−kLNaiveLSelf−kLNaiveLMat−>I−kLNaiveLMat−>A)],

where the remaining indices instead of (24) are given by

(26) kLNaiveLSelf=−μLSelf−νLSelf+kLSelf,kLNaiveLMat->I=−μLMat->I−νLMat->I+kLMat->I,kLNaiveLMat->A=μLNaive+μLSelf+μLMat->I−νLNaive−νLSelf−νLMat->I−kLSelf−kLMat->I,kLMat->A=μLNaive+μLSelf+μLMat->I+μLMat->A−νLNaive−νLSelf−νLMat->I−νLMat->A−kLSelf−kLMat->I.

The selection phase of lymphocytes is described by

(27) WnS(NLNaive,NLSelf,NLMat->I,NLMat->A)=∑νLNaive=NLNaiveNmax⁡∑νLSelf=NLSelfNmax⁡−νLNaive ∑νLMat->I=NLMat->INmax⁡−νLNaive−νLSelf ∑νLMat->A=NLMat->ANmax⁡−νLNaive−νLSelf−νLMat->I WnM(νLNaive,νLSelf,νLMat->I,νLMat->A) ·(νLNaiveNLNaive)βLNaiveNLNaive(1−βLNaive)(νLNaive−NLNaive) ·(νLSelfNLSelf)βLSelfNLSelf(1−βLSelf )(νLSelf−NLSelf) ·(νLMat->INLMat->I)βLMat->INLMat->I(1−βLMat->I)(νLMat->I−NLMat->I) ·(νLMat->ANLMat->A)βLMat->ANLMat->A(1−βLMat->A)(νLMat->A−NLMat->A).

## 5. Discussion and Conclusion 

Understanding the dynamics of both an invading pathogen and the response of the host's immune system is an essential task in one's attempt to positively influence the immune response of the given host. However, one already experiences difficulties in modeling the behavior of a single biological cell. A cell (as an element of a population of such cells) divides more frequently within a favorable environment and may form new strains by occasional errors in the copying process. It also dies more probably within a less favorable environment. 

How should one model such a step-by-step Darwinian process? Some may opt for numerically solving a set of first order differential equations, where time is continuous, and then examine the mainly nonlinear properties of variables (which represent large population rescaling). In contrast, we presented here a simple stochastic model of an entity undergoing a continued step-by-step Darwinian process, which is subdivided into two phases of multiplication (with variation) and selection. We describe this stochastic mathematically by a recursion formula (Galton-Watson type) for each phase, the dynamics of the system being evaluated numerically by computer, where the number of generation is an integer time-variable. The form of probability distribution *W*(*N*) changes in this system dynamics (with *N* being the number of individuals, and including the probability *W*(0) of extinction). This is a great advantage of this approach.

In addition, at a more fundamental level, one can suggest the following experiment to verify the Galton-Watson type dynamical stochastic process without mutation, described by equations (3) and (4) resulting in Figure 4 (case Section 2.3), or with copying errors leading to one mutant, described by equations (9)–(12) resulting in Figure 7 (case Section 2.4) and its parameter range: one prepares a steady-state condition of a bacterium-culture on a growing medium, where an antibiotic is added to the nutrient solution in a sublethal concentration, by repeated consecutive single-cell inoculation procedures. Then one can count bacteria by stopwatch the final single-cell inoculations carried out in parallel with the same nutrient solution (case Section 2.3) or with the nutrient solution charged additionally with another antibiotic of sub-lethal concentration (case Section 2.4) and plot the resultant time-dependant histogram.

In this paper, we studied three types of behavior by analyzing both the pathogen and the host's immune reaction with the proposed model system: (i) lasting pathogen elimination with buildup of immune memory, (ii) an oscillatory chronic case, where the pathogen is almost eliminated by the activated immune system, while during the subsequent relaxation of the immune system the pathogen is flaring up again, and (iii) the two-strain case, where the pathogen can vary its antigen at one epitope resulting in a prolonged immune-response.

In order to map such a simple mathematical model of the immune-response to a real system, for example, a specific host, a specific pathogen, and a specific pathway, further work should consider the particular properties (e.g., the relative doubling rate) of the pathogen and the particular properties of the T and B lymphocytes and other host properties as done by the aforementioned authors [2–4]. Explicitly considering genotype and phenotype should also be fruitful. Finally, one can find possible applications of the model in HIV, LCMV, influenza virus, herpes virus, mycobacterium tuberculosis, and plasmodium or trypanosomes. Supplementary material provided on the Website of CMMM available online at doi:10.1155/2012/784512.

## Supplementary Material

The Supplementary material includes videos (Video 3, Video 4, Video 7, Video 9, Video 10, Video 12, Video 13) demonstrating the time development from which snap-shots were singlet out and displayed in the corresponding figures (see inside PDF) of the paper *Immune Response to a Variable Pathogen: A Stochastic Model with Two Interlocked Darwinian Entities* by Christoph Kuhn.
Video 3: Dynamical stochastic process of multiplication and selection. Discrete limit.
Video 4: Dynamical stochastic process of multiplication and selection. Continuous limit.
Video 7: Dynamical stochastic process for initial form *A* and mutant *B*.
Video 9: Computer result of a pathogen *P* (with antigen *A*) coupled through averages to an immune system consisting of an effector *E* (producing antigen receptor *a*) and memory *M*. Case where pathogen is eliminated.
Video 10: Computer result of a pathogen *P* (with antigen *A*) coupled through averages to an immune system consisting of an effector *E* (producing antigen receptor *a*) and memory *M*. Case where the pathogen is reappearing while the immune system response is low.
Video 12: Computer result of a varying pathogen (*P_A_* with antigen *A* changing into *P_B_* with antigen *B*) coupled through averages to an immune system against antigen *A* consisting of an effector *E_a_* and memory *M_a_* and against antigen *B* consisting of an effector *E_b_* and memory *M_b_*. Partial change of pathogen *P_A_* to pathogen *P_B_* escaping immune effector *E_a_* and with delayed immune response of effector *E_b_*.
Figure 13: Computer result of the maturation process of T-lymphocytes. First a naive T-lymphocyte (*L*_Naive_, green) in bone marrow or thymus undergoes T-cell receptor rearrangement (*β*-selection). T-cells with high affinity to self-peptides MHC (*L*_Self_, black) are eliminated (negative selection), whereas T-cells with T cell receptors that are able to bind self-peptides MHC molecules with at least a weak affinity (*L*_Mat->*I*_, blue and *L*_Mat->*A*_, red) survive (positive selection) and circulate in the peripheral lymphatic system. The matured T-lymphocyte, recognizing the antigen by high affinity to the antigen-loaded MHC (*L*_Mat->*A*_, red), transforms into an effector cell and proliferates.
